# Suitability of MDA, 8-OHdG and wild-type p53 as genotoxic biomarkers in metal (Co, Ni and Cr) exposed dental technicians: a cross-sectional study

**DOI:** 10.1186/s12903-020-1049-1

**Published:** 2020-03-06

**Authors:** Titiek Berniyanti, Retno Palupi, Indah L. Kriswandini, Taufan Bramantoro, Indira L. Putri

**Affiliations:** 1 Department of Dental Public Health, Faculty of Dental Medicine, Universitas Airlangga, Surabaya, Indonesia; 2 Department of Biology Oral, Faculty of Dental Medicine, Universitas Airlangga, Surabaya, Indonesia

**Keywords:** Dental technician, Toxicity, Heavy metal, 8-OHdG, MDA, p53, Oxidative damage

## Abstract

**Background:**

High concentrations of Co, Ni, and Cr in the blood serum of dental technicians are strongly associated with free radical formation. It has highly reactive properties that can cause further oxidation of molecule in the vicinity.

**Purpose:**

This study intended to investigate whether the Dental Technician occupational exposure of Co, Ni and Cr, could contribute to the high incidence of cancer.

**Methods:**

This was a cross-sectional study to dental technicians, performed after acccepting ethical clearance. Blood was sampled in 3 examinations for Co, Ni, Cr using Atomic Absorbance Spectrophotometry (AAS), MDA was examined with TBARS test, also 8 OHdG and wildtype p53 proteins determined by ELISA method.

**Results:**

Comparative statistical analysis, showing a significant difference (*p* < 0.05) between levels of Co, Ni, and Cr in exposed groups to the control group. But, not all variables was proven to be positively correlated, only with Cr, and Co, and negatively correlated with wild-type p53.

**Conclusion:**

MDA,8-OHdG and wildtype p53 can be used as genotoxic biomarkers in the metal exposed group, since they can accurately reflect the degree of Oxidative damage.

## Introduction

The alloys Cobalt-chromium (Co-Cr) and Nickel-Chromium (Ni-Cr) are used extensively in dental medicine for removable partial dentures, porcelain-fused-to-metal crowns, and metal frames and particularly because they are, as compared to gold, bio-compatible, have a remarkable strength, corrosive resistant, and relatively less costly [1].. However, along with the benefits, during the manufacturing process, metal alloy exposure, as well as inaccurate working conditions and protection of workers, exposure to metals is hazardous to worker health. As more and more evidence found on metal-based nanoparticles in carcinogenicity, more studies have emerged, aimed to evaluate those nanoparticles’ genotoxicity and carcinogenicity, especially copper, chromium, nickel, and cobalt. The studies included those on epigenetic factors, for example, increased oxidative stress, abnormal apoptosis, and pro-inflammatory effects that involve those nanoparticles [2]..

The study, conducted by Hariyani et al., proved the impact of metal exposure significantly, ie the concentration of metal in the blood of dental technicians is higher than the control through research entitled, the effect of controlling the work environment on the levels of Co, Ni and Cr in the blood of the dental technicians [3]. The high concentration of Co, Ni and Cr is strongly associated with the formation of free radicals. It is highly reactive properties that can cause further oxidation of molecule in the vicinity. DNA damage due to ROS and DNA bases modifications results in altered genomic information. The damage may include deletions, insertions, point mutations, or chromosomal translocations that may lead to the oncogene activation as well as tumor suppressor gene inactivation, which have potential for initiating carcinogenesis [4]..

Reaction between ROS and membrane lipids can easily occur at high levels. This results in changes of membrane permeability, which results in chain reaction where a component of the cell membrane, the unsaturated fatty acid, is oxidized within varied pathological conditions. This condition is called as lipid peroxidation. The attack of radicals on polyunsaturated fatty acid residues of phospholipids leads to the formation of lipid peroxides, which furthermore may induce reaction with redox metals, and finally producing mutagenic and carcinogenic malondialdehyde, 4-hydroxynonenal and other exocyclic DNA adducts (etheno and/or propano adducts). As chromium (Cr) and cobalt (Co) perform redox-cycling reactions, nickel (Ni), a second group of metals, have the primary route of toxicity through glutathione depletion and the binding to sulfhydryl groups of proteins [5].. Oxidative attack on lipids leads produces reactive aldehydes as well. Among several different aldehydes that can be formed as secondary products of lipid peroxidation, MDA is the most mutagenic product of lipid peroxidation [6].

ROS react with DNA molecules causes modifications or damage to DNA structure and genomic instability affects genetic information contained inside. 8-OHdG is particularly a ROS-induced DNA base modification resulting from the attack of Hydroxyl radicals (OH) to guanine which triggers damage to the DNA. If it is not repaired, the damage will also be involved in cancer promotion and mutagenicity. Among the various types of oxidative DNA damage, 8-hydroxydeoxy guanosine (8-OHdG) formation is the most suitable marker of oxidative stress everywhere [7]. When DNA damage occurs, p53 protein activate s the p21 gene and the GADD45 gene for DNA repair. When DNA damage was severe, p53 protein activates the gene to trigger apoptotic processes [8]. If the cells cannot make improvements, there will be a disruption of DNA repair and result in cell death (pyknosis, karyorrhexis, karyolysis) [9].

ROS reaction with proteins results in oxidative modifications that causes catalytically less active enzymes or proteins that have higher susceptibility to proteolytic degradation. Protein damage may take place with carbonylation, thiol oxidation, fragmentation, side-chain oxidation, unfolding and misfolding, which may finally lead to activity loss [10]. In this case ROS are detrimental and induce cell apoptosis or necrosis. However, when the production of ROS does not change cell viability irreversibly, ROS may serve as primary messenger that modulates some cascades of intracellular signaling that leads to the progress of the cancer [5]..

The objective of this study was to review the suitability of MDA, 8-OHdG and p53 as Genotoxic Biomarkers, of Co, Ni and Cr dental technicians exposed in the workplace in contributing to the high incidence of cancer from metal exposure in the workplace.

## Methods

### Sample of the study

The participants of this study were 40 individuals working as the dental technicians who worked on the metal prosthesis for at least 3 years in Dental Laboratory Surabaya, Indonesia. Participant information sheet was given, and a signed consent form was obtained from the participants prior collecting blood samples.

### Blood samples collecting and processing

As much as 6 m blood was drawn from anticubital vein by a professional nurse of all the 40 individuals working as the dental technicians who worked on the metal prosthesis for at least 3 years in Dental Laboratory Surabaya, in Indonesia by means of sterile disposable syringes after surface sterilization of the skin area with alcohol. Blood sampling was carried out in the morning, at 7–9 am, so that the body basal state can be obtained since the daily activity have not begun.

Four milliliters of total blood sample without anticoagulant content was preserved for serum separation was obtained to study of MDA and 8 OHdG. Similarly, 2 ml of total blood sample containing anticoagulant was obtained to analyze metal concentration: Co, Ni, and Cr. Samples that had been collected were preserved in screwed bottles with identification slips. The samples were brought to the laboratory in an ice container, not more than 3 h.

### Measurement of human blood co, Ni, and Cr level

Blood samples were collected in the tubes with EDTA. Metal level measurement was performed at Regional Health Laboratory, Surabaya, Indonesia, by using Atomic Absorbance Spectrophotometry (AAS) in a wavelength of 240.7 nm, 232.0 nm and 357.9 nm. The unit of measurement results were in μg/L.

### Measurement of serum MDA levels

MDA was defined as the product of lipid peroxidation which is able to react to thiobarbituric acid to give the red species absorb at 535 nm. Procedure: one ml of patient serum is added to 9 ml of cold PBS, and 4 ml is taken from the supernatant. The supernatant solution was combined with 2 ml of 15% Trichloroacetic acid (TCA) - 1 ml 0.37% Thiobarbituric acid (TBA) solution - in 0.25 N Hydrochloric acid (HCL) solution, mixed thoroughly, then heated in water bath up to 800 C for 15 mins, and cooled at room temperature for 60 min. After cooling, centrifugation is done at 3000 rpm for 10 min to remove the precipitate. The absorbance is determined at 535 nm against an empty reagent, which contains all of the reagents minus the serum. The absorbance of the MDA supernatant sample was measured on a spectrophotometer at λ = 532 nm. The MDA analysis was calculated using the regression equation of the standard MDA (standard) solution curve.

### Measurement of 8-OHdG level

Evaluation of 8-OHdG concentrations in the serum used commercial test of the OxiSelect™ DNA Oxidative Damage ELISA Kit was used (Cat No. STA-320, Cell Biolabs, Inc., USA). One hundred ml 8-OHdG / BSA conjugate is added to a 96-well plate, and the incubation was carried out overnight at 4 °C, and then being washed with H2O. The followin step was the addition of 200 μl blocking buffer and incubation was done at room temperature for 1 h. Then we added as much as 50 μl samples and 8-OHdG standard.

Monoclonal anti-8-OHdG was subsequently added as much as 100 μl for an hour incubation at room temperature. After incubation for 10 min, it was washed three times, then added with conjugated secondary antibody to peroxidase radishes as much as 100 μl for 1 h incubation at room temperature. The plate was washed three times with a washer and 100 μl peroxidase substrate was added to each hole and the incubation was carried out for 20 min, followed by the addition of 100 μl reaction cessation solution. The measurement of spectrophotometric absorbance was done in a wavelength λ = 450 nm. 8-OHdG content in the tested sample was calculated by comparison with standard curve determined from the standard treated in similar way as the tested sample.

### Measurement of p53 level

The expression of Wild-type p53 protein was measured from saliva and analyzed by indirect ELISA methods. The protein p53 is conserved much the evolution, whose expression can found in most normal tissues. Wild-type protein p53 is known to serve as a sequence-specific transcription factor, which has direct interaction with many cellular and viral proteins. Subjects were instructed not to eat, not smoke, and not rinse with antiseptics two hours before saliva was taken. Subsequent saliva samples were taken between 10:00 and 13:00 [11], by allowing the saliva to accumulate then ordered to spit into the tube. Then all collected saliva was centrifuged 3000 g for 15 min at 4oC until the supernatant was obtained [12].

Furthermore, the supernatant was analyzed for its Wild-type p53 protein by the indirectly ELISA method using Human p53 ELISA Kit (ab171571) (p53 protein tumor) (Elabscience Biotechnology Co., Wuhan, Hubei, China). Double-stranded oligonucleotides contained p53 consensus. The DNA binding sequence was incubated with pure TP53 protein dilution, and TP53 bound to oligo was catched onto the microtiter plate surface. After being washed, TP53 bound was detected with an anti-p53 primary antibody, tumor protein p53, and followed by an HRP-labeled secondary antibody. After the development of the initial stain, the reaction was quenched and the stain intensity was measured at 450 nm. All of these experiments were performed at the Institute of Tropical Disease, Universitas Airlangga.

### Measurement of personal protection equipment (PPE)

The assessment of PPE covers the frequency and manner of the use of masks, gloves, eyeglasses, laboratory work clothes, and shoes by dental technicians, which are based on the category of exposure of the main metal pathway that enters the body (Personal Protective quetionnaire assessment is attached as “Suplementary File”). Masks and gloves have the highest percentage because metal exposure enters the body through three main channels; breathing, mouth and skin [12]. The categories of frequency of PPE use are divided into: always, rarely, and never (100, 50, and 0). The category scores of PPE use procedures are divided into true, incorrect, and incorrect scores of 100, 50, and 0. The final score is obtained by summing the APD usage scores of frequency and the PPE score usage procedure. The score is multiplied by the percentage of PPE weight. The percentage of weighing is 30% for masks, 25% gloves, 20% for glasses, 15% for lab work clothes and 10% for shoes.

## Result

**Fig. 1:**
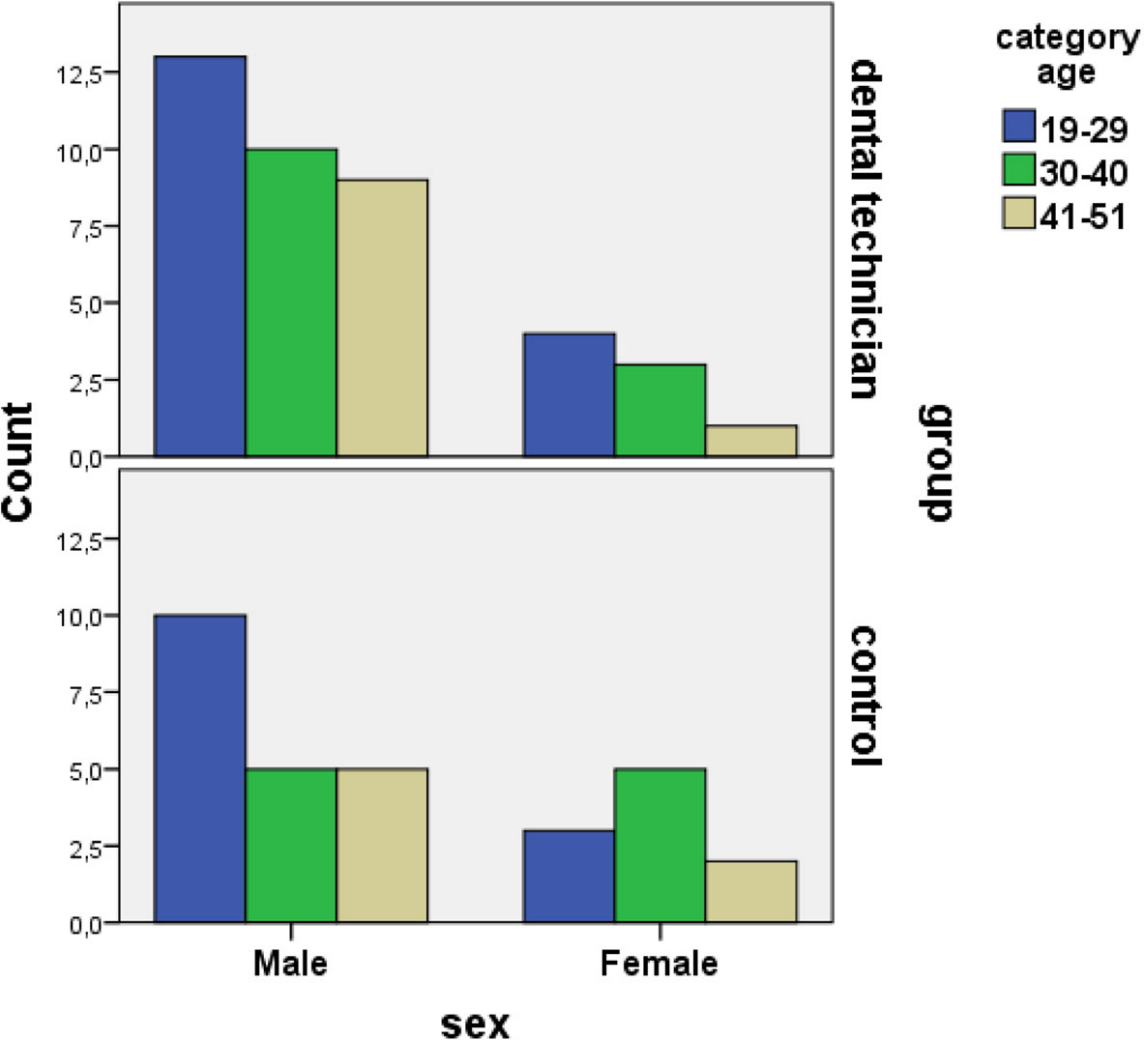
Age and Range Based on Gender

Dental technicians that participated in this study were mostly in population of 19–29 years old or as much as 42.5% of the samples, while those of the age group 41–51 years were only 25%. Majortiy of the respondents were male, while women included in this study were few. (Fig. 1). The Cr concentration in dental technicians was the highest compare with Co and Ni concentration in dental technicians. Mean value of MDA and 8-OHdG in dental technicians were higher than controls. Comparative test revealed a significant difference with *p* value 0.000 (*p* < 0.05) between the values of the metal, MDA and 8-OHdG levels in the blood of the technicians as compared to the value of the metal in control.

There is significant correlation between 8- OHdG and heavy metals. The same result was also recorded on MDA and heavy metals. The observation revealed that Ni showed very weak correlation with both 8-OHdG (r = 0.06) and MDA (r = 0.09). Meanwhile, Co and Cr showed weak correlation with both 8-OHdG (Co: r = 0.337; Cr: r = 0.355) and MDA (Co: r = 0.337; Cr: r = 0.306). For PPE variable, 8-OHdG showed negative correlation (r = − 0.04) which means the higher 8-OHDG concentration, the less PPE be used (Table 1).

**Table 1:** Mean, 95% confidence interval, and *p*-Value of each variable according to result of comparison test between exposed and control group

Variable (μg/l)	Group	Mean	95%CI	*p*-Value
Cobalt				
	Exposed	26,75	14,78 – 38,72	0,000
	Controls	0,43	0,37 – 0,50	
Nickel				
	Exposed	36,76	21,96 – 51,56	0,000
	Controls	3,35	2,90 -3,81	
Chromium				
	Exposed	346,36	303,78–388,94	0,000
	Controls	0,06	0,03 – 0,08	
8-OHdG				
	Exposed	6,31	5,41 – 7,21	0,000
	Controls	0,87	0,76 - 0,99	
MDA				
	Exposed	8,34	6,96 – 9,71	0,000
	Controls	0,39	0,31 - 0,47	
Wildtype p53				
	Exposed	0,27	0,18 – 0,36	0,000
	Controls	0,58	0,50 – 0,65	

The correlation between heavy metal level and MDA concentration is illustrated in Fig. 1. Age and Range Based on Gender. This study involved a total sample of 40 respondents (32 males and 8 females), and 30 respondents belonged to control group with 18 males and 12 females. The sample group consisted of 17 respondents aged 19–29 years old (13 males and 4 females), 13 respondents aged 30–40 years old (10 males and 3 females), 10 respondents aged 41–51 years old (9 males and 1 females). The control group were dominated with respondents aged 19–29 years old (13 respondents with 10 males and 3 females), followed by 30–40 years old (10 respondents with 5 males and 5 females), and 41–51 years old (7 respondents with 5 males and 2 females). The following data were considered a balance proportion for both group.

**Fig. 2:**
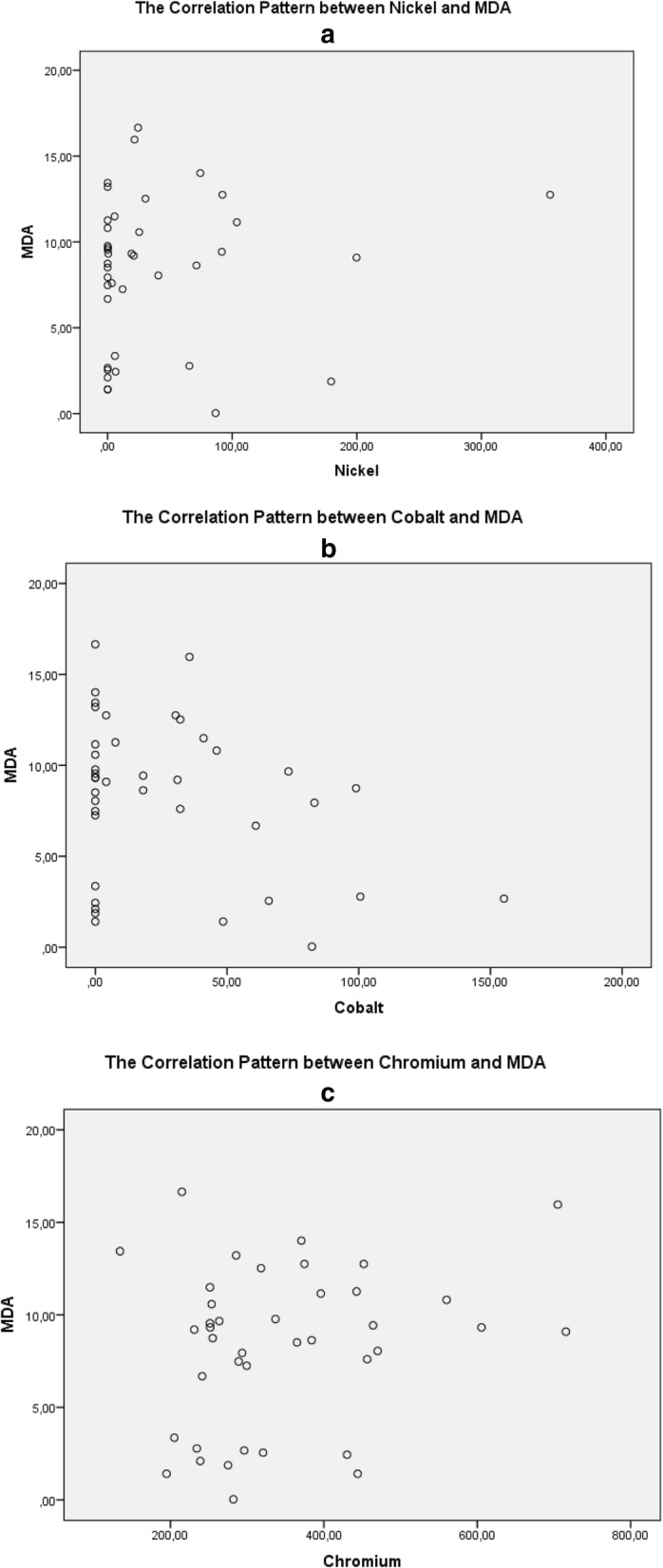
shows the patterns of relationship between Heavy Metal (Ni, Co, Cr) and MDA. Figure 2**a** shows the pattern of relationship between Nickel and MDA, 2**b** shows the pattern of relationship between Cobalt and MDA, and Figure 2**c** shows the pattern of relationship between Chromium and MDA

Figure 2a shows the pattern of the relationship between Nickel and MDA. The higher the level of nickel, the higher the level of MDA. Figure 2c shows the pattern of the relationship between Chromium levels and MDA levels. The higher the level of Chromium, the higher the level of MDA. Thus the levels of Nickel and Chromium are positively related to MDA levels. While Fig. 2b shows an inverse relationship between Cobalt levels and MDA levels, the higher the Cobalt level, the lower the MDA level.

**Fig. 3:**
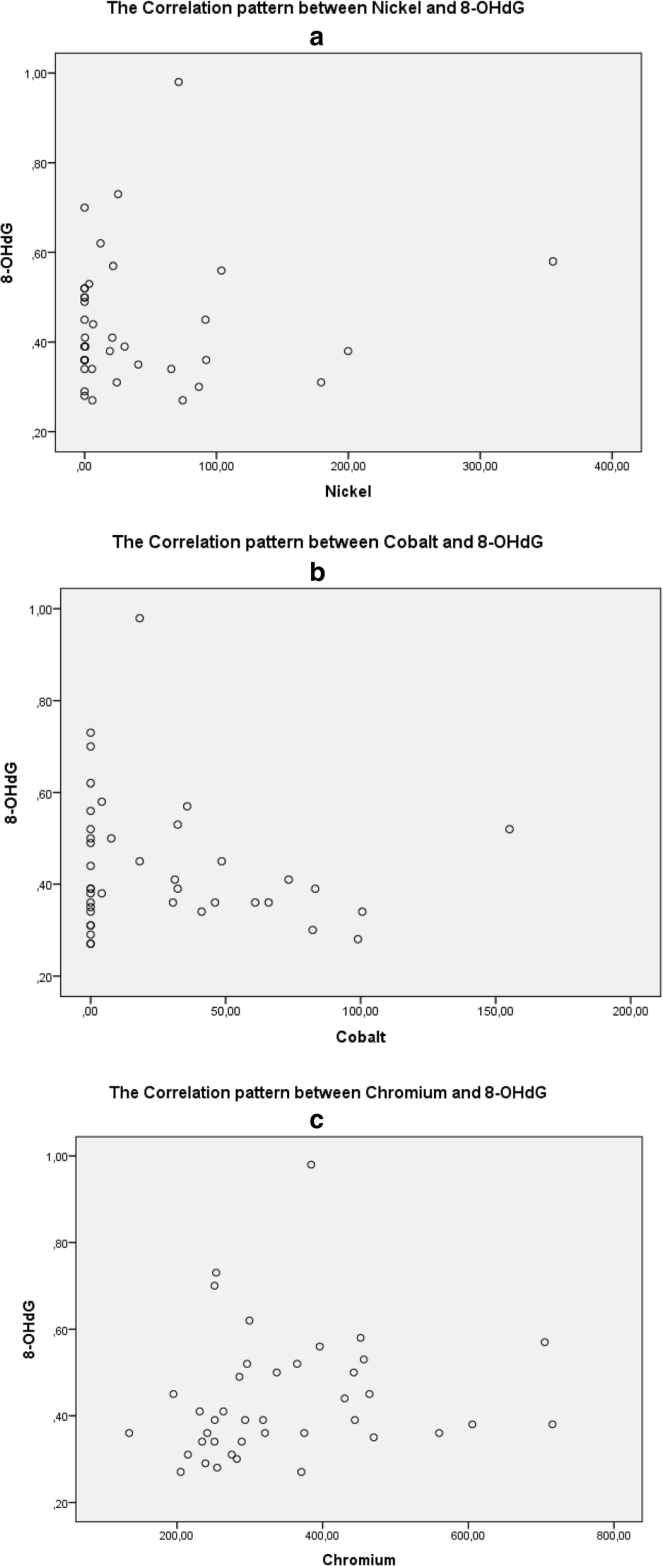
shows the pattern of relationship between Heavy Metal (Ni, Co, Cr) and 8-OHdG. **a** shows the pattern of relationship between Nickel and 8-OHdG, **b** shows the pattern of relationship between Cobalt and 8-OHdG, and **c** shows the pattern of relationship between Chromium and 8-OHdG

Figure 3 shows the higher levels of metals (Nickel, Cobalt, Chromium), the higher the level of 8-OHdG. A positive relationship was established between Nickel levels and 8-OHdG levels, between Cobalt and 8-OHdG levels, as well as between Chromium and 8-OHdG levels.

**Fig. 4:**
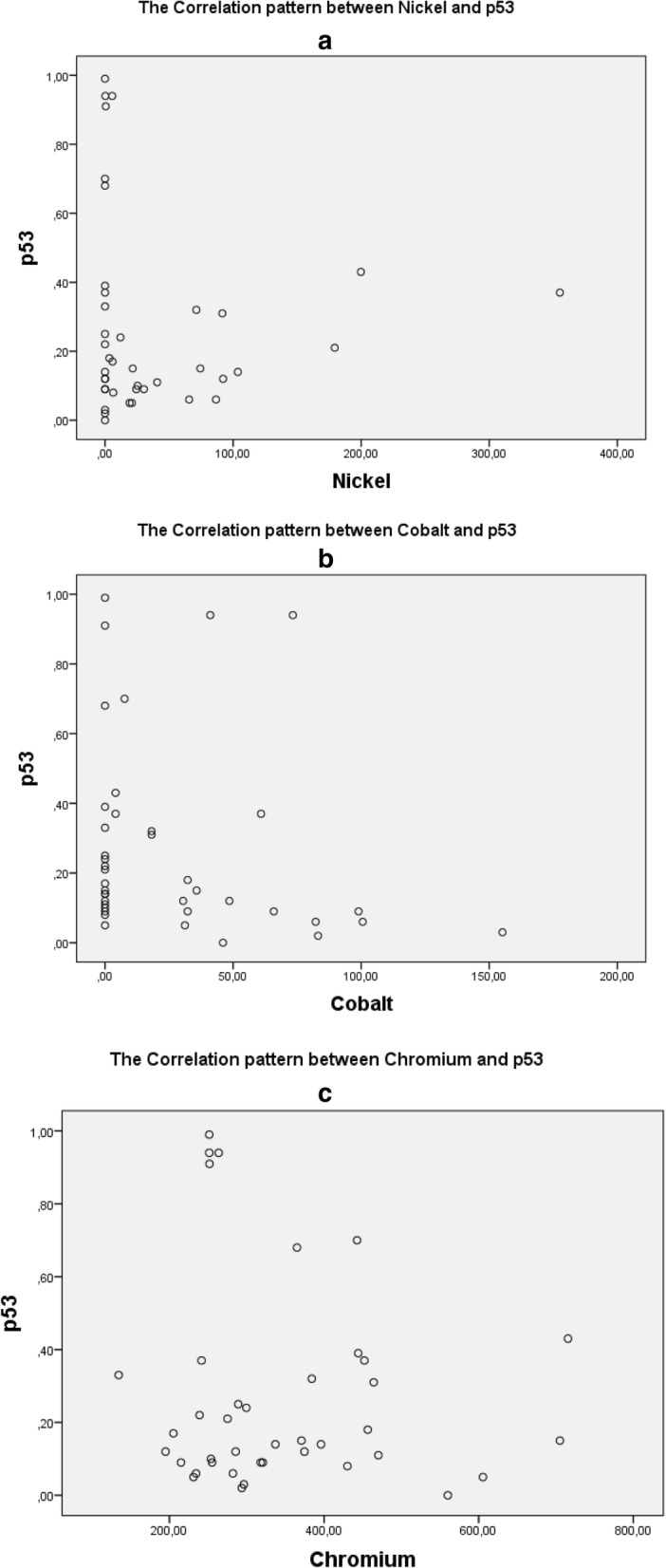
shows the patterns of relationship between Heavy Metal (Ni, Co, Cr) and p53. **a** shows the pattern of relationship between Nickel and p53, **b** shows the pattern of relationship between Cobalt and p53, and **c** shows the pattern of relationship between Chromium and p53

**Fig. 5:**
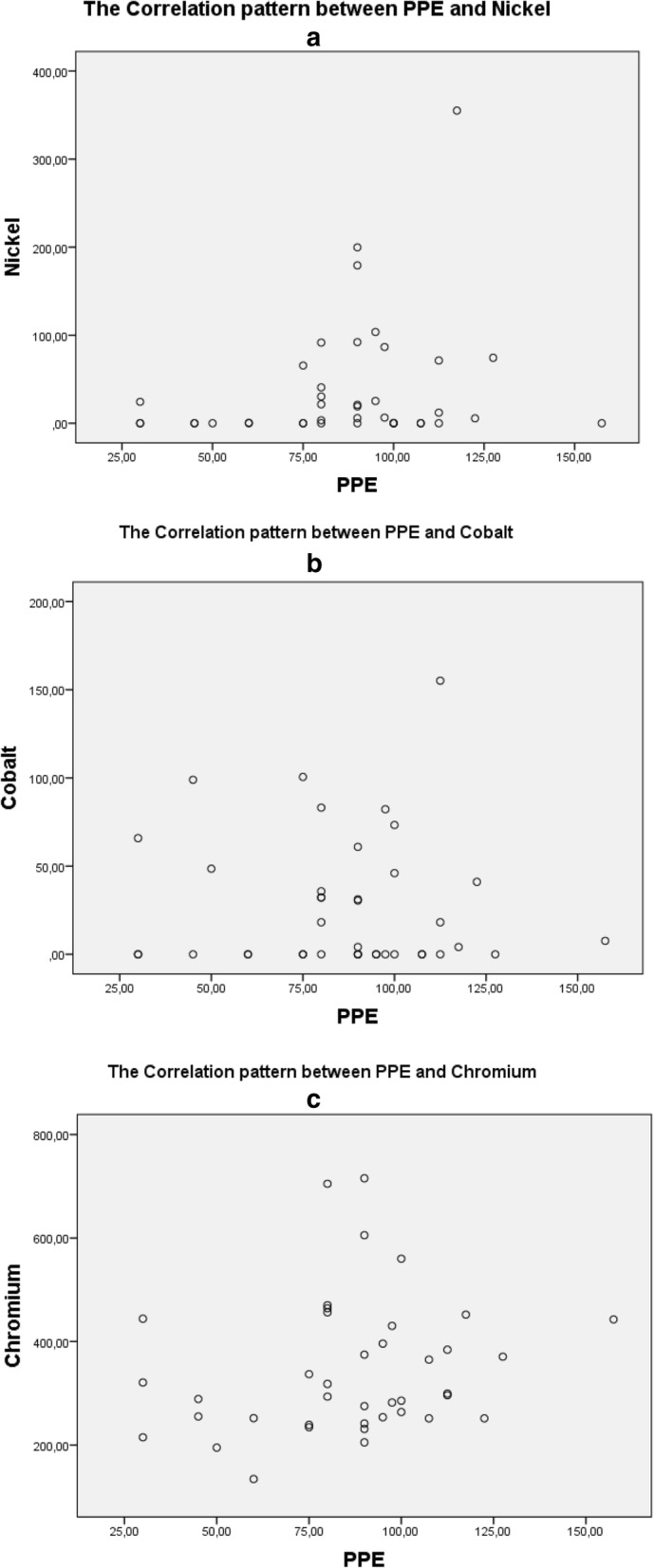
shows the patterns of relationship between PPE and Heavy Metal (Ni, Co, Cr). **a** shows the pattern of relationship between PPE and Nickel, **b** shows the pattern of relationship between PPE and Cobalt, and **c** shows the pattern of relationship between PPE and Chromium

Figure 4 contains the correlation pattern between heavy metals and p53. It is stated that a high level of Chromium may also increase p53 level in blood. Meanwhile, a negative correlation was recorded for Cobalt; the higher Cobalt level, the less p53 expressed. A positive correlation also found between PPE with both Chromium and Nickel, yet interestingly, PPE and Cobalt level is showing a negative correlation (Fig. 5). Figure 5 shows pattern of relationship between metal content and PPE. It shows a positive correlation between PPE and metal (Cr and Ni), and a negative correlation for PPE and Co.

**Fig. 6:**
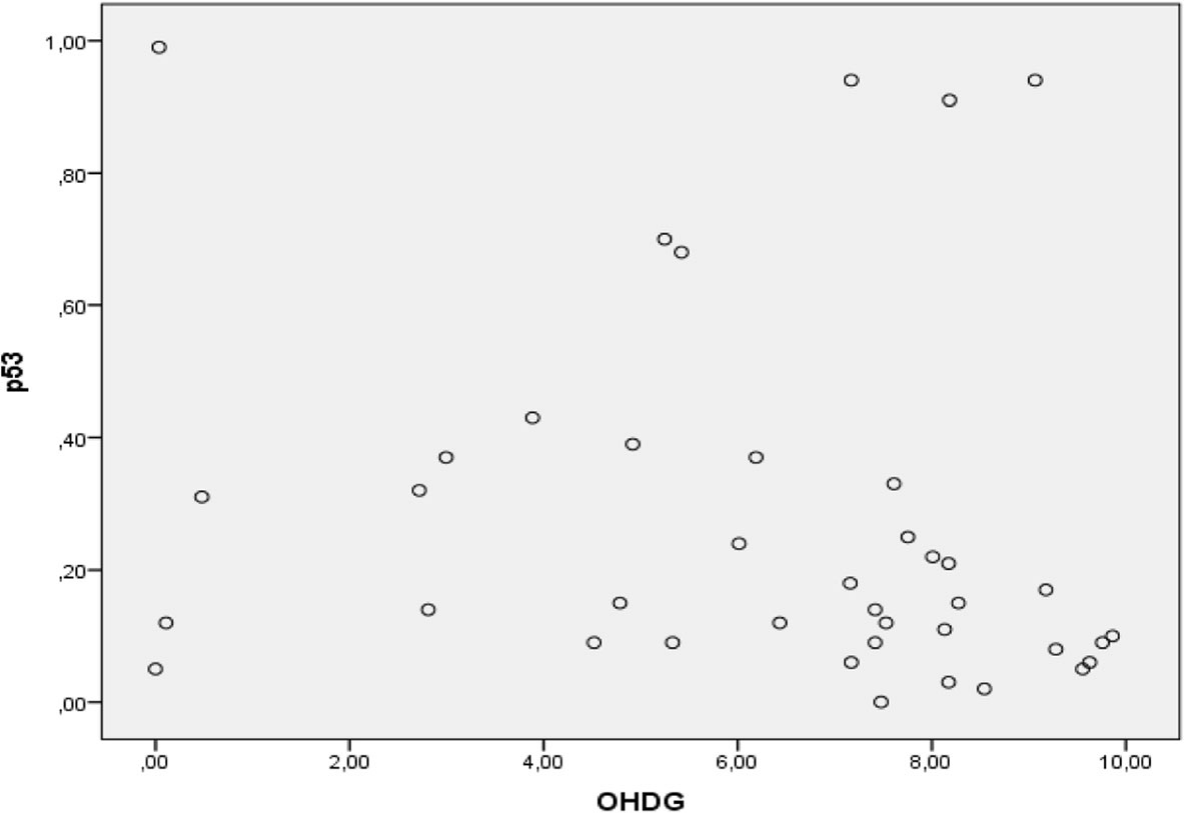
above shows the correlation pattern between 8-OHdG and p53

Figure 6 shows that the correlation pattern between 8-OHdG and p53 is negative. If 8-OHdG level is high, then the level of p53 is high. Conversely, if 8-OHdG level is high, p53 levels are low.

Table 1 show Mean, 95% confidence interval, and *p*-Value of each variable according to result of comparison test between exposed and control group. There are significant differences of metal concentration (Ni, Co, Cr) between the dental technicians and the control group. There are significant differences of MDA concentration between the dental technicians and the control group. There are significant differences of 8OHdG concentration between the dental technicians and the control group. There are significant differences of wild type p53 concentration between the dental technicians and the control group.

## Discussion

Previous research has concluded that dental technicians at Dental Laboratory in Surabaya have high Co, Ni and Cr levels. The high levels of metals in the blood of the dental technicians are due to the lack of use of self-protective devices so that exposure to metals can be absorbed through inhalation and skin during the manufacture of dental prostheses such as crowns, bridges and framework of partially released dentures [3]. Some of metal compounds have carcinogenic characteristics to human and animals as well. Metal-mediated free-radicals formation induces a variety of modifications to DNA bases, enhances lipid peroxidation, as well as alters the calcium and sulfhydryl homeostasis.

Cr actually plays role in human body to help the metabolism of glucose and fat. Cr exposure produces remarkable effect on human immune system, and, therefore, normally it is advised that exposure of the workers to chromic acid in electroplating industries should be reduced up to a minimum level. Co plays a role in the catalysis of vitamin B12 and is essential to the vitamin’s biological activity [13]. Ni. has an important role in iron absorption process in the body. However, nickel (Ni) is also reported as exerting intoxication to industrial workers. The risk of respiratory cancer is high in some certain groups workers who are exposed ro nickel, even though not all forms of Ni exposure are involved in such excessive risks [14].. In physiological conditions, these three metals are required only in very small quantities. The recommended reference values ​​are 0.09–4.18 μg / L for Ni, 0.04–0.64 μg / L for Co. and 0.1–0.2 μg / L for Cr [15]. The toxicity of Nickel, Cobalt, and Chromium is associated with its oxidation form. Cr (III), Cr (VI), Ni (II) is the most common and stable form of oxidation of Cr and Ni and is categorized as group 1 carcinogen associated with the incidence of lung cancer. Co. can also be found in oxidation forms of Co (II) and Co (III) and grouped as 2B group carcinogens.

This oxidation form acts as a catalyst in Fenton-like and Haber-weiss reactions to enhance Reactive Oxygen Species (ROS) formation in the form of hydroxyl radicals (OH) [16, 17]. Increased levels of metals can increase the process of free radical formation resulting in an imbalance between intracellular ROS and antioxidants and gives rise to a state of oxidative stress [18]. Increased production of ROS may worsen the dysfunction of mitochondria, in part due to peroxidation (oxidative damage of the lipids) [19].. Lipid hydroperoxides was produced through the reaction between free radical and double bonds of polyunsaturated fatty acids (PUFAs). It is a chain reaction from which free radicals supply were continuously provided due to its involvement in polyunsaturated fatty acid oxidation in the membrane that leads to damaged oxidative cells [20]. Malondialdehyde (MDA), a major secondary oxidation product of peroxidized polyunsaturated fatty acids, was believed to induced cytotoxic and mutagenic effects and might be related to cell aging and has a role in the onset of chronic morbidities such as cancer, atherosclerosis, inflammation, etc. [6, 21]. In this study statistical data obtained from the determination of MDA on serum dental technicians showed the Mean and SD higher than control.

Increased levels of oxidative stress in serum Dental technicians in this case represent an increase in local free radical production [21]. This indicated the activity of free radicals in cells as one of the clues of oxidative stress. Comparative statistical analysis between Co, Ni, and Cr levels in the exposed group with the control group, MDA, in blood serum of dental technicians showed significant differences (*p* < 0.05). This phenomenon showed that dental technician is one of the high risk occupations due to hazardous metal exposure. This high risk was indicated by the MDA value, where in normal individuals is less than 1.03 nmol/ml, and 2 times of that MDA value was considered pathologic. There is an important role that free radicals have in various diseases [22]..

When carcinogenic compounds entered the body and were activated, this compound would have covalent interactions with DNA to form DNA-adduct (modified DNA). This interaction could also lead to the breaking of the DNA chain. Among whole photooxidative DNA products, the biomarker 8-hydroxy-deoxyguanosine (8-OHdG) is sensitive and stable in the evaluation of DNA damage degree [23].. In our study the comparative statistical analysis between the levels of Co, Ni, and Cr in the group exposed to the control group, 8-OHdG, and wild type p53 protein in blood serum from dental technicians all showed a significant difference (*p* < 0.05). Although 8-OHdG level was not all proven to be positively correlated, only with Cr, and Co, and were negatively correlated with wild type p53, the comparative tests have shown the effect of high metal (Co, Ni and Cr) on the interactions with DNA to form DNA adducts, or 8-OHdG ensured that there has been damage to the DNA [24].

Associated with the analysis carried out on wild type p53 proteins, because p53 is considered a protein that has a function in the induction of apoptosis or arresting the cell cycle as a response to DNA damage, so that it maintains genetic stability of the organism. p53 shows genes that have the most mutations, especially in malignancies. Through the development of molecular biology at this time, it was revealed that one of the factors causing malignancy was the failure of the tumor suppressor gene, p53.

Physiologically if DNA damage occurs due to oxidative stress, the wild type p53 protein activates p21 and the gene for Catching Growth and Protein Inducible Protein Damage (GADD45) to enable DNA repair. Wild type p53 protein levels will increase, and play a role in DNA repair. If DNA damage is severe, cells will undergo apoptosis [25], or a p53 gene mutation occurs, which causes mutant p53 proteins to increase. In accordance with the research, it appears in this study that wild type p53 protein levels were lower than wild type p53 protein levels in controls and p53 negatively correlated with 8 OHdG. Decrease in wild p53 protein levels is caused by mutations in p53. Mutant p53 protein has a negative dominant effect on wild type p53 proteins by suppressing the wild-type p53 protein, which would result in the inactivation of p53 protein function. So there is a decreasing in wild-type p53 protein levels, and increasing in mutant levels of p53. Mutation of the p53 gene is believed to be associated with the result [26]. This finding supports the existing theory that p53 mutants have a negative predominant effect on wild type p53 protein, so if there is a decrease in wild p53 levels there may be an increase in p53 mutant levels [27, 28].

The oxidative damage also might affect the cell cycle, lead to mutations, and produce carcinogenesis because of enzymatic, endogenous, and nutritional antioxidant systems failure [29, 30]. According to Matsui, 8-OHdG played an important role in the early phase of carcinogenesis [31]. Enzyme couldn’t be able to recognize DNA molecules and trigger the formation of uncontrol growth cell that lead into malignancy. Oxidative DNA damage was mediated by reactive oxygen species and played an vital role in the occurrence of some diseases, including cancer [6]. With early detection of the risk, DNA damage was be prevented further so it did not cause cancer [32].

## Conclusion

MDA, 8-OHdG and wild-type p53 can be used as genotoxic biomarkers in the metal exposed group, since they can accurately reflect the degree of oxidative damage.

## Supplementary information

**Additional file 1.** Personal Protective Equipment Assessment Questionnaire.

## Data Availability

The data analyzed during the current study are not publicly available. They are available from the corresponding author on reasonable request.

## Abbreviations

8 OHdG: 8-hydroxydeoxyguanosine
AAS: Atomic absorbance spectrophotometry
Co: Cobalt
Cr: Chromium
DNA: Deoxyribonucleic acid
GADD45: The growth arrest and DNA damage-inducible 45
HCL: Hydrochloric acid
MDA: Malondialdehyde
Ni: Nickel
p21 gene: The cyclin-dependent kinase inhibitor p21
ROS: Reactive oxygen species
TBARS test: Thiobarbituric acid reactive substances
TCA: Trichloroacetic acid
wildtype p53: The p53 tumor suppressor protein
