# Supplementary material for: Suitability of MDA, 8-OHdG and wild-type p53 as genotoxic biomarkers in metal (Co, Ni and Cr) exposed dental technicians: a cross-sectional study
Source: BMC Oral Health. 2020 Mar 6;20:65. doi: 10.1186/s12903-020-1049-1 (PMC7059730; doi:10.1186/s12903-020-1049-1)
Supplement: Supplementary file 1 — Additional file 1. Personal Protective Equipment Assessment Questionnaire. [file 12903_2020_1049_MOESM1_ESM.docx]

**SUPLEMENTARY FILE 1: PERSONAL PROTECTIVE EQUIPMENT ASSESSMENT QUESTIONNAIRE**

Table 2

**Assessment score for the use of PPE**

| **Frequency of PPE usage** | **Score** |
| --- | --- |
| Always | 100 |
| Rarely | 50 |
| Never | 0 |
| **How to use PPE** | **Score** |
| Right | 100 |
| Not all Right | 50 |
| Wrong | 0 |

**Percentage weight of PPE usage behavior**

a. Masks : 30%

b. Gloves : 25%

c. Protective goggles : 20%

d. Lab coat : 15%

e. Shoes : 10 %

**Put a check mark (√) in one of the columns provided.**

Information : A : Always (score 100)

R : Rarely (score 50)

N : Never (skor 0)

**List of Questions for PPE quesionner.**

| **Questions** | | **A** | **R** | **N** |  |
| --- | --- | --- | --- | --- | --- |
| 1. | Change lab coats that are worn every day |  |  |  |  |
| 2. | Lab coats are used only when working in a dental laboratory |  |  |  |  |
| 3. | Lab coats use the operator according to body size |  |  |  |  |
| 4. | The average operator only uses one mask in one day |  |  |  |  |
| 5. | Mask used to cover the mouth and nose |  |  |  |  |
| 6. | Gloves are used according to the size of the hand |  |  |  |  |
| 7. | When the glove used is hole or torn, it is immediately replaced |  |  |  |  |
| 8. | Washing hands after removing gloves |  |  |  |  |
| 9. | Use disposable gloves |  |  |  |  |
| 10. | When you want to touch personal items (cellphone, wallet, stationery, etc.), take off your gloves first then wash your hands |  |  |  |  |
| 11. | Eating or drinking while still using gloves |  |  |  |  |
| 12. | Wear and remove gloves in the right way |  |  |  |  |
| 13. | Check the condition of the glove before wearing it |  |  |  |  |
| 14. | Wear shoes while working according to size |  |  |  |  |
| 15. | Use shoes to work that covers the entire foot |  |  |  |  |
| 16. | Special shoes are only used when working in a dental laboratory |  |  |  |  |
| 17. | Use protective glasses that cover all parts of the eye when working |  |  |  |  |
| 18. | Have a smoking habit |  |  |  |  |
| 19. | Number of cigarettes in a day | 0 | 1-10 | 11-20 | >20 |

**Discription of Data**

The behavior of PPE usage in dental technicians who work daily with chemicals in the Surabaya dental laboratory was assessed based on the frequency of PPE usage and the procedures for using PPE. The PPE used consisted of masks, gloves, protective goggles, lab coats, and shoes.

When someone wears 3 PPE such as a mask, gloves, and lab coat while another person also wears 3 PPE but the type of PPE is different, such as masks, protective goggles, and shoes. So, of the two people, which one has better PPE usage behavior? Of course this can not be determined because the two people are both using 3 types of PPE. Therefore, the weighting method is used to assess the behavior of PPE usage. The weight of the percentage of the use of PPE masks, gloves, protective glasses, laboratory coats, and shoes are respectively 30%, 25%, 20%, 15%, and 10% so that the percentage when added together results in a 100% figure.

Masks and gloves have the highest percentage, given that dental chemicals enter the body through three main channels, namely the respiratory, oral, and skin (Costa, 2002). Therefore, masks have the highest percentage because the use of appropriate masks can prevent the entry of chemical exposure through the respiratory tract as well as through oral. According to Kundi et al (2010), efficient use of suitable masks can also reduce exposure to inhaled chemicals reaching 70% -95%. Meanwhile, gloves have the second largest percentage because the use of gloves can prevent exposure to chemicals that enter through dermal exposure.

Furthermore, the third highest percentage is occupied by protective glasses. That is because as long as dental technicians work with chemicals, the eye organ is one of the organs on the face that has the closest distance to a product being worked on. Although the eye organ is not one of the first routes of chemical entry into the body, based on the results of the study, 35% of dental technicians have eye disorders and technicians who wear protective eye glasses have a 7% lower incidence of eye disorders than those who do not use protective eye glasses (Yurdasal et al, 2015).

Then, the fourth highest percentage is occupied by protective clothing (laboratory coats). Laboratory coats are used with the aim of preventing the deposition of chemical exposures that can stick to the clothes of dental technicians. The lowest percentage is occupied by shoes. The use of shoes aims to protect the feet when liquid chemicals are spilled.

Frequency of PPE usage consists of always, rarely, and never, with each value ranging from 100, 50, and 0. The greater the value the higher the frequency of PPE usage, the better it is. Likewise, the procedures for using PPE that consist of true, not always true, and false. Each of these scores is 100, 50, and 0. The higher the score, the more correct the usage method. The score results are multiplied by each percentage of PPE. Later, the frequency score for the use of each PPE is summed with the score for how to use each PPE. All scores obtained by the study subjects are added up and averaged and the scores are divided into two.
